# Lower vascular conductance responses to handgrip exercise are improved following acute antioxidant supplementation in young individuals with post‐traumatic stress disorder

**DOI:** 10.1113/EP091762

**Published:** 2024-05-06

**Authors:** Jennifer B. Weggen, Ashley M. Darling, Aaron S. Autler, Austin C. Hogwood, Kevin P. Decker, Jacob Richardson, Gina Tuzzolo, Ryan S. Garten

**Affiliations:** ^1^ Department of Kinesiology and Health Sciences Virginia Commonwealth University Richmond Virginia USA; ^2^ Department of Kinesiology University of Texas at Arlington Arlington Texas USA; ^3^ Department of Kinesiology and Applied Physiology University of Delaware Newark Delaware USA; ^4^ Department of Kinesiology University of Virginia Charlottesville Virginia USA

**Keywords:** antioxidant, autonomic nervous system, mental health, vascular function

## Abstract

Young individuals with post‐traumatic stress disorder (PTSD) display peripheral vascular and autonomic nervous system dysfunction, two factors potentially stemming from a redox imbalance. It is currently unclear if these aforementioned factors, observed at rest, alter peripheral haemodynamic responses to exercise in this population. This study examined haemodynamic responses to handgrip exercise in young individuals with PTSD following acute antioxidant (AO) supplementation. Thirteen young individuals with PTSD (age 23 ± 3 years), and 13 age‐ and sex‐matched controls (CTRL) participated in the study. Exercise‐induced changes to arm blood flow (BF), mean arterial pressure (MAP) and vascular conductance (VC) were evaluated across two workloads of rhythmic handgrip exercise (3 and 6 kg). The PTSD group participated in two visits, consuming either a placebo (PL) or AO prior to their visits. The PTSD group demonstrated significantly lower VC (*P *= 0.04) across all exercise workloads (vs. CTRL), which was significantly improved following AO supplementation. In the PTSD group, AO supplementation improved VC in participants possessing the lowest VC responses to handgrip exercise, with AO supplementation significantly improving VC responses (3 and 6 kg: *P *< 0.01) by blunting elevated exercise‐induced MAP responses (3 kg: *P* = 0.01; 6 kg: *P *< 0.01). Lower VC responses during handgrip exercise were improved following AO supplementation in young individuals with PTSD. AO supplementation was associated with a blunting of exercise‐induced MAP responses in individuals with PTSD displaying elevated MAP responses. This study revealed that young individuals with PTSD exhibit abnormal, peripherally mediated exercise responses that may be linked to a redox imbalance.

## Abstract (highlights)

- **What is the central question of this study?**
  Do young individuals with post‐traumatic stress disorder (PTSD) display abnormal peripheral haemodynamic responses during exercise?
- **What is the main finding and its importance?**
  Young individuals with PTSD displayed lower vascular conductance (VC) during handgrip exercise compared to healthy counterparts. Antioxidant supplementation notably improved VC responses, especially in the subset of PTSD participants with the highest pressor responses during exercise. This study underscores abnormal peripherally mediated exercise responses in young individuals with PTSD, suggesting a potential link to a redox imbalance.

## 1 INTRODUCTION

Post‐traumatic stress disorder (PTSD) is a debilitating stress‐related psychological disorder (American Psychiatric Association, 2013; Watson, 2019) that is widely prevalent among the general population, documented in approximately 8–11 million individuals in a given year (Goldstein et al., 2016; Schein et al., 2021). Although psychological in nature, PTSD is accompanied by physiological manifestations which impose an increased risk in developing cardiovascular disease (CVD). Indeed, individuals with PTSD report greater levels of CVD risk factors such as hypertension (Kibler et al., 2009), as well as a higher prevalence of CVD development specific to heart failure (Roy et al., 2015), coronary atherosclerosis (Ahmadi et al., 2011), myocardial infarction (Remch et al., 2018), coronary heart disease (Kubzansky et al., 2009) and stroke (Remch et al., 2018). Numerous negative lifestyle behaviours (e.g., physical inactivity, smoking, alcohol consumption, poor diet, illicit drug use) are also linked to PTSD, resulting in a higher prevalence of modifiable CVD risk factors such as obesity (Pagoto et al., 2012), diabetes (Lukaschek et al., 2013) and metabolic syndrome (Rosenbaum et al., 2015). Investigating young adults with PTSD prior to overt disease development better clarifies the mechanisms by which PTSD increases CVD risk, negating or minimizing the confounding effects imposed by these negative lifestyle behaviours.

An abnormal blood pressure response to exercise is a major characteristic of accelerated CVD development (Jae et al., 2015; Manolio et al., 1994), and likely occurs secondary to dysfunction of the peripheral vasculature and/or dysregulation of the autonomic nervous system (Mitchell, 2017). In healthy individuals, blood flow to the skeletal muscle is tightly regulated to ensure adequate oxygen delivery to meet the metabolic demands of the muscle (Joyner & Casey, 2015), with the peripheral vasculature and autonomic nervous system playing critical roles in this regulation (Joyner & Casey, 2015). Dysregulation of skeletal muscle blood flow can result from local dysfunction of the microvasculature due to an imbalance in dilatory and constrictor vasoactive substances, and/or elevated sympathetic nervous system (SNS) activation/transduction, all of which can result in excessive vasoconstriction within the working muscle (Mitchell, 2017). Prior work by our laboratory identified microvascular dysfunction in the arm and leg as well as autonomic nervous system imbalance in young (∼23 years) individuals with PTSD (Weggen et al., 2021), but as these measures were employed at rest it is unclear if these consequences of PTSD are robust enough to impact blood flow regulation during exercise, a hallmark characteristic of future hypertension and CVD development (Jae et al., 2015; Yang et al., 2014). Recent work has revealed elevated SNS activation/transduction in response to isometric exercise in middle‐aged individuals with PTSD (D'Souza et al., 2022), highlighting abnormal peripherally mediated mean arterial pressure (MAP) responses to exercise, but it is unknown if this response is present in young individuals or if this abnormal response is present during exercise with a distinct contraction–relaxation pattern (e.g., isotonic), which more directly translates to normal and/or occupational activities of daily living. Of interest, an acute antioxidant supplementation restored both upper limb microvascular function and autonomic nervous system balance at rest in young individuals with PTSD to that of well‐matched controls (Weggen et al., 2021), potentially implicating the consequences of redox imbalance as a mediator of this dysfunction. Indeed, redox imbalance, or the state to which oxidant production overwhelms the exogenous and endogenous antioxidant defence within the body, has been implicated as a major by‐product of the psychological symptoms of PTSD (Hori & Kim, 2019; Miller & Sadeh, 2014) and as a mediator of peripheral vascular dysfunction (Greaney et al., 2019; Wray et al., 2017) and SNS overactivation (Grotle & Stone, 2019).

Collectively, peripheral microvascular dysfunction and SNS overactivation can impact the normal blood flow and MAP response to exercise. Determining whether such dysfunction/dysregulation would be robust enough to alter peripherally mediated exercise responses in young individuals with PTSD requires further investigation. This study employed small muscle mass exercise (rhythmic handgrip exercise), a practical method of assessing vascular function (Wray et al., 2011) and skeletal muscle‐derived SNS activation, to determine if blood flow, mean arterial pressure and vascular conductance responses to exercise are altered in young individuals with PTSD. We hypothesized that young individuals with PTSD would exhibit an elevated pressor response and lower blood flow/vascular conductance during handgrip exercise when compared to well‐matched controls. This would provide evidence of an abnormal exercise response derived from peripherally mediated mechanisms (microvascular dysfunction, SNS overactivation) in this population. To determine the potential role of redox imbalance in abnormal PTSD‐induced exercise response, we utilized acute antioxidant supplementation to investigate its effect on exercise responses in individuals with PTSD. We hypothesized that following acute antioxidant (AO) supplementation, pressor and blood flow/vascular conductance responses would be improved, implicating redox imbalance as a mediator of this dysfunction/dysregulation.

## 2 METHODS

### 2.1 Ethical approval

All study procedures were approved by the Institutional Review Board of Virginia Commonwealth University (HM20009929), and written informed consent was attained according to their guidelines. This study was performed to the standards set by the *Declaration of Helsinki*, except for registration in a database.

### 2.2 Subjects

Thirteen young, otherwise healthy adults with PTSD (nine female, four male) and 13 sex‐ and age‐matched healthy controls were recruited from Virginia Commonwealth University and the Richmond, Virginia metropolitan area to participate in this study. The PTSD group comprised the same participants who participated in a recently published study that identified microvascular dysfunction and SNS predominance at rest in this population (vs. healthy controls) (Weggen et al., 2021). Apart from subject demographics, no other data have been previously published. All participants provided written informed consent, in accordance with institutional guidelines as approved by the Institutional Review Board of Virginia Commonwealth University. Everyone completed a medical history questionnaire, with no reports of overt cardiovascular, metabolic or lung disease. All participants stated that they were free from illicit drug use, substance abuse and tobacco use. Only those with PTSD were consuming prescribed medication at the time, as indicated in Table 1, and their medication regimen was not altered during this study. Physical activity was collected from each participant, based on minutes per day spent being physically active. Those who self‐reported PTSD were given the PTSD Checklist for DSM‐5 (PCL‐5) to confirm eligibility. The sole criterion used for inclusion in the PTSD group was a positive PTSD screening with a PCL‐5 score of >30 (Bovin et al., 2016; Geier et al., 2019). The PCL‐5 was selected for its strong reliability and validity, and is used for screening of PTSD, provisional diagnosis and research (Bovin et al., 2016; Geier et al., 2019). The PCL‐5 correlates highly with the Clinician‐Administered PTSD Scale (CAPS‐5), which is considered the gold standard in PTSD assessment tools (Bovin et al., 2016; Geier et al., 2019). All data from the healthy control group were acquired from prior data collection (unpublished) where participants had completed the handgrip exercise protocol described below, under identical conditions, during a single visit for which neither the antioxidant supplementation nor placebo was consumed. Although 35 healthy controls were included in our laboratory's prior publication (Weggen et al., 2021), only 15 of the 35 healthy controls (9F/6M) completed the handgrip exercise protocol. Thus, matching the healthy controls to the PTSD group was based solely on age and biological sex with two males excluded to match participant numbers between groups. The exclusion of the two males was based on neither having ages that identically matched the four male PTSD participants. Body composition measurements (seven‐site skinfold), height and weight were acquired at the end of the session for this group, and at the end of the first session for the PTSD group. Moderate and vigorous physical activity for each subject (self‐report) was collected through a medical history questionnaire.

**TABLE 1:** Subject characteristics.

Characteristic	CTRL (*n* = 13)	PTSD (*n* = 13)
Sex (female/male)	9/4	9/4
Age (years)	23 ± 3	23 ± 4
Height (cm)	171 ± 12	168 ± 10
Weight (kg)	69 ± 16	71 ± 21
Body fat (%)	21 ± 11	26 ± 10
PCL‐5 score (a.u.)	—	47 ± 8
Moderate/vigorous daily physical activity (min)	39 ± 13	38 ± 15
Handgrip maximum workload achieved (kg)	9.0 ± 2.7	9.5 ± 2.7
Medications (*n* (%))		
Selective serotonin reuptake inhibitor	0 (0)	4 (31)
Anxiolytic	0 (0)	3 (23)
Serotonin‐noradrenaline reuptake inhibitor	0 (0)	2 (15)
Alpha‐adrenergic blocker	0 (0)	2 (15)
Anti‐convulsant	0 (0)	2 (15)
Serotonin receptor antagonist and reuptake inhibitor	0 (0)	1 (8)
Noradrenaline and dopamine reuptake inhibitor	0 (0)	1 (8)
Guanylate cyclase‐C agonist	0 (0)	1 (8)
Anti‐cholinergic	0 (0)	1 (8)
Anti‐psychotic	0 (0)	1 (8)
β‐Adrenergic blocker	0 (0)	1 (8)
Thyroid replacement	0 (0)	1 (8)
Testosterone replacement	0 (0)	1 (8)
CNS stimulant	0 (0)	1 (8)
Corticosteroid	0 (0)	1 (8)

*Note*: Data displayed as means ± standard deviation. Abbreviations: a.u., arbitrary units; CTRL, control group; PTSD, PTSD group.

### 2.3 Study protocol

Individuals with PTSD completed two study visits with similar protocols, receiving either microcrystalline cellulose placebo pills (PTSD‐PL), or antioxidants (PTSD‐AO) (500 mg of vitamin C, 400 milligrams (mg) alpha lipoic acid, and 200 mg vitamin E) (Nature Made, West Hills, CA, USA), which were consumed in the stated amount twice, first at 120 min prior to the study visit and then again 90 min prior to the study visit to ensure plasma antioxidant levels were at their highest (Donato et al., 2010; Richardson et al., 2007). The antioxidant cocktail was selected for its ability to decrease free radical concentration in plasma (Donato et al., 2010; Ratchford et al., 2019; Rossman et al., 2013; Wray et al., 2011), which can enhance bioavailability of vasodilatory molecules in healthy and diseased populations (Donato et al., 2010; Ratchford et al., 2019; Rossman et al., 2013). The two sessions were double‐blinded, randomized and at least 72 h apart to allow adequate washout of the antioxidants from the body (Ives et al., 2013; Wray et al., 2009). Females in the PTSD group (*n* = 9) were not limited to a specific phase of their menstrual cycle. This was relevant in eight female subjects, as the remaining female subject had an intrauterine device and did not menstruate. All female control participants were tested during the first 7 days of their menstrual cycle or during the placebo phase of oral hormonal contraceptive use (*n* = 1).

All participants arrived for their testing session having abstained from food for at least 6 h, caffeine for 12 h, and exercise and alcohol consumption for at least 24 h prior to testing. All sessions took place in a quiet, climate‐controlled room at a similar time of day.

The testing protocol consisted of multiple 3 min dynamic, progressive, rhythmic handgrip exercise bouts completed in the supine position with the participant's right arm. Participants started at the 3 kg workload which was performed for 3 min followed by a 3 min rest period. This exercise–rest pattern continued with workloads increasing in 3 kg intervals (e.g., 6, 9 kg) until participants could not complete the full 3 min workload. During each exercise workload, participants rapidly squeezed the handgrip dynamometer (TSD121C; Biopac Systems, Goleta, CA, USA) once every second (1 Hz) timed with a metronome, while following real‐time force tracings on a computer monitor (Biopac Systems) to which a kilogram threshold (e.g., 3, 6, 9 kg) was achieved during each contraction. Brachial artery blood flow (BA BF), heart rate (HR) and MAP (Tango M2; SunTech Medical, Inc., Morrisville, NC, USA) were obtained at rest and during the final minute of each workload, when steady state was achieved. All workloads were separated by a 3 min rest period. The handgrip protocol was completed following four other vascular/autonomic assessments (heart rate variability, brachial and superficial artery flow mediated dilatation, passive leg movement) as previously described (Weggen et al., 2021), with adequate rest periods (>10 min) occurring between each vascular assessment. Specific to the handgrip exercise protocol, participants rested in a supine position for 15 min prior to baseline measures.

### 2.4 Peripheral haemodynamic measures

Brachial artery diameter and blood velocity were acquired via Doppler ultrasound technology (LOGIQe Doppler ultrasound system; GE Healthcare, Milwaukee, WI, USA), utilizing a linear array transducer operating at an imaging frequency of 14 MHz. Both diameter imaging and velocity measures were obtained simultaneously with the probe appropriately placed over the brachial artery, centred between the axillary and antecubital regions, maintaining an insonation angle of 60° or less. Maximum sample volume was set according to the size of the blood vessel and centred within the lumen of the vessel using real‐time ultrasound visualization. Careful attention to probe placement was noted by using anatomical landmarks for accurate repeat assessments.

Ultrasound images of the brachial artery were analysed after the study visit to ascertain the absolute (mm) change in diameter, using automated edge detection software (Medical Imaging Applications, Coralville, IA, USA). BA BF in millilitres per minute (π × *V*_m_ × (arterial diameter/2)^2^ × 60) and shear rate in s^−1^ (8 × *V*_m_/arterial diameter) were calculated using the mean blood velocity (*V*_m_) (angle corrected and intensity weighted) values. Vascular conductance (VC), at rest and during exercise, was calculated as brachial artery blood flow divided by MAP.

### 2.5 Statistical analysis

Student's *t*‐test for independent samples was used to examine differences between groups in participants’ demographic data (Table 1). A one‐way analysis of variance (ANOVA) was employed to determine differences in resting values for BA diameter, BA BF, BA shear rate, HR, MAP, VC between groups/conditions (PTSD‐PL vs. PTSD‐AO vs. CTRL). In order to attain a complete data set, the 3 and 6 kg workloads were utilized for data analysis as they represented the only two workloads that all PTSD and CTRL participants were able to successfully accomplish. Significant differences between groups (PTSD‐PL vs. PTSD‐AO vs. CTRL) and across workloads were explored using a mixed model analysis of variance (RMANOVA) for absolute and change from baseline (Δ) values of MAP, heart rate, BA diameter, BA BF, BA shear rate and VC. For statistical analysis of the mixed model ANOVA, the Bonferroni correction method was implemented to reduce the risk of type I error, and simple main effects were explored if any significant interactions were present. Additional mixed model ANOVAs were utilized with maximum achieved handgrip workload as a covariate to determine if relative (vs. absolute) workloads explained any significant differences identified between groups. For the exploratory analysis in individuals with PTSD only, a median cut point for VC responses at the 3 and 6 kg workloads was employed to separate groups into either low (3 kg: ≤1.0 mL min^−1^ mmHg^−1^; 6 kg: ≤1.5 mL min^−1^ mmHg^−1^; *n* = 7) or high (3 kg: >1.0 mL min^−1^ mmHg^−1^; 6 kg: >1.5 mL min^−1^ mmHg^−1^; *n* = 6) exercise‐induced VC responses. A RMANOVA was then utilized to determine potential interaction effects of the AO supplementation (vs. PL) within each group for VC, MAP and BF responses at the 3 and 6 kg workloads. Sample size was calculated based on prior literature published by our laboratory (Weggen et al., 2023) examining group differences in VC during handgrip exercise as well as prior literature reporting improvements in exercise‐induced BF and VC responses following acute AO supplementation in older adults (Carlson et al., 2008) and evaluated with two‐tail directionality at α = 0.05, power (1 − β) of 0.8, and effect size of 1.2 and 0.9, respectively, using SPSS Statistics 25.0 (IBM Corp., Armonk, NY, USA), which indicated a total of 12 participants would be required for this study. Normal data distribution was assessed via the Shapiro–Wilk test. SPSS 25.0 was used for all analysis with statistical significance set at *P* ≤ 0.05. Data are presented as means ± standard deviation.

## 3 RESULTS

Comparison of participant characteristics presented in Table 1 revealed no significant differences in age (*P =* 0.92), height (*P =* 0.49), weight (*P =* 0.79), body fat percentage (*P =* 0.21), daily moderate‐to‐vigorous physical activity (*P =* 0.56) or maximum workload achieved during handgrip exercise (*P =* 0.67) between the PTSD and CTRL groups (Table 1). No differences in central or peripheral haemodynamic measures at baseline were present between groups/conditions (Table 2).

**TABLE 2:** Baseline central and peripheral haemodynamic measures.

Variable	CTRL	PTSD‐PL	PTSD‐AO	*P*
Heart rate (bpm)	66 ± 9	67 ± 12	68 ± 12	0.88	
Mean arterial pressure (mmHg)	82 ± 3	87 ± 6	86 ± 6	0.06	
BA diameter (mm)	3.76 ± 0.73	3.71 ± 0.75	3.77 ± 0.76	0.97	
BA shear rate (s^−1^)	145 ± 57	141 ± 78	139 ± 77	0.98	
BA blood flow (mL min^−1^)	45 ± 25	38 ± 12	38 ± 15	0.46	
Vascular conductance (mL min^−1^ mmHg^−1^)	0.56 ± 0.29	0.53 ± 0.35	0.45 ± 0.19	0.60	

*Note*: Data displayed as means ± standard deviation. Abbreviations: bpm, beats per minute; CTRL, control group; PTSD‐AO, PTSD group antioxidant condition; PTSD‐PL, PTSD group placebo condition.

**FIGURE 1:**
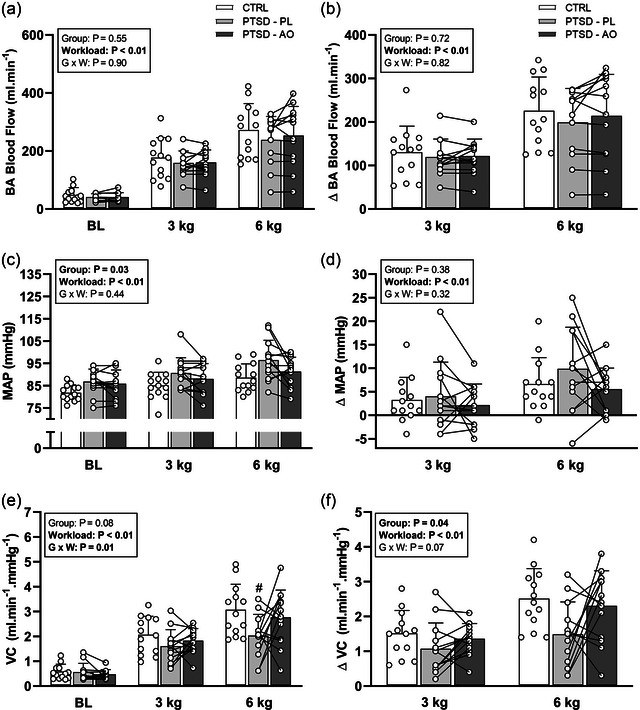
Change in brachial artery (BA) blood flow (BA blood flow: a; ΔBA blood flow: b), mean arterial pressure (MAP: c; ΔMAP: d), and vascular conductance (VC: e; ΔVC: f) in response to HG exercise at 3 and 6 kg in young healthy controls (CTRL) and individuals with posttraumatic stress disorder after acute consumption of placebo (PTSD+PL) and antioxidants (PTSD+AO). Data are represented as means ± standard deviation. Data were evaluated with two‐way repeated measures analysis of variance. #*P < *0.05 versus control group at the same workload.

During handgrip exercise, a main effect of workload for both groups was revealed with significant increases in BA diameter, BA shear rate, BA BF, MAP, VC and HR with increasing workloads (Table 3 and Figure 1) when examined as both absolute values (BL < 3 kg < 6 kg) and change from baseline (3 kg < 6 kg). No significant group or interaction effects were identified when examining in absolute or change from baseline values for BA diameter, BA shear rate and HR (Table 3).

**TABLE 3:** Central and peripheral haemodynamics during handgrip exercise.

	CTRL	PTSD‐PL	PTSD‐AO	*P*
HR (bpm)				G = 0.96 WL < 0.01 G × WL = 0.78
BL	66 ± 9	67 ± 12	68 ± 12	
3 kg	69 ± 11	69 ± 13	69 ± 12	
6 kg	73 ± 11	72 ± 14	74 ± 12	
ΔHR (bpm)				G = 0.60 WL < 0.01 G × WL = 0.74
3 kg	3 ± 6	2 ± 5	2 ± 5	
6 kg	7 ± 5	5 ± 7	6 ± 6	
BA diameter (mm)				G = 0.94 WL < 0.01 G × WL = 0.91
BL	3.76 ± 0.73	3.71 ± 0.75	3.77 ± 0.76	
3 kg	3.91 ± 0.64	3.80 ± 0.69	3.87 ± 0.72	
6 kg	4.09 ± 0.72	3.99 ± 0.67	4.10 ± 0.77	
ΔBA diameter (mm)				G = 0.60 WL < 0.01 G × WL = 0.82
3 kg	0.15 ± 0.21	0.09 ± 0.13	0.09 ± 0.13	
6 kg	0.33 ± 0.22	0.28 ± 0.21	0.33 ± 0.30	
BA shear rate (s^−1^)				G = 0.94 WL < 0.01 G × WL = 0.92
BL	145 ± 57	141 ± 78	139 ± 77	
3 kg	517 ± 242	527 ± 205	507 ± 192	
6 kg	725 ± 315	694 ± 317	655 ± 286	
ΔBA shear rate (s^−1^)				G = 0.72 WL < 0.01 G × WL = 0.74
3 kg	372 ± 217	386 ± 172	368 ± 155	
6 kg	580 ± 295	553 ± 302	516 ± 263	

*Note*: Data displayed as means ± standard deviation. Abbreviations: bpm, beats per minute; CTRL, control group; G, group main effect; G × WL, group × workload interaction effect; PTSD‐AO, PTSD group antioxidant visit; PTSD‐PL, PTSD group placebo visit; WL, workload main effect.

BA BF, examined as both absolute and change from baseline, revealed no significant main effect of group or group × workload interaction (Figure 1a, b). A significant group main effect was revealed for absolute MAP (Figure 1c) with a *post hoc* analysis of absolute MAP response to handgrip exercise across groups/conditions revealing a significant difference between the CTRL and PTSD‐PL groups (*P =* 0.01), but no significant difference between the CTRL and PTSD‐AO groups (*P =* 0.52) or the PTSD‐PL and PTSD‐AO conditions (*P =* 0.06) (Figure 1c). When evaluated as ΔMAP to examine pressor responses to handgrip exercise independent of baseline differences, no significant group or interaction effects were observed.

No group main effect, but a significant interaction effect (group × workload) was observed for absolute VC revealing a significant difference between the CTRL and PTSD‐PL groups (*P =* 0.01) at the 6 kg workload only (Figure 1d). When a change from baseline (ΔVC) was evaluated to examine the haemodynamic response to handgrip exercise independent of baseline values, a significant group main effect was revealed with a *post hoc* analysis identifying a significant difference between the CTRL and PTSD‐PL groups (*P =* 0.01), but no significant difference between the CTRL and PTSD‐AO groups (*P =* 0.52) or the PTSD‐PL and PTSD‐AO conditions (*P =* 0.06) (Figure 1f). No significant group or interaction effects were observed. No significant group differences were seen in maximum handgrip workload achieved (Table 1), and with this factor employed as a covariate, the analysis of the outcome variables did not alter the results discussed above.

**FIGURE 2:**
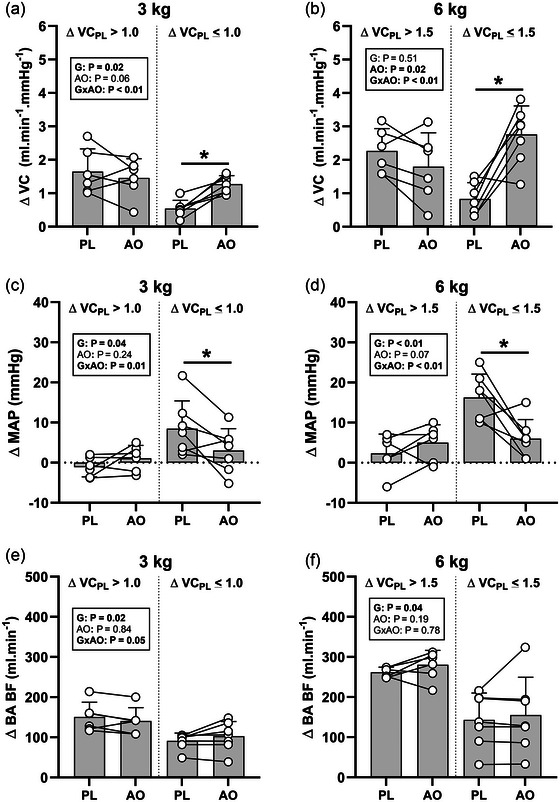
Change in vascular conductance (ΔVC; a, b), mean arterial pressure (ΔMAP; c, d), and brachial artery blood flow (ΔBF; e, f) in response to 3 and 6 kg handgrip workloads during the PL and AO visit in young individuals with PTSD separated by low (3 kg: ≤1.0; 6 kg: ≤1.5 mL min^−1^ mmHg^−1^; *n* = 7) or high (3 kg: >1.0; 6 kg: >1.5 mL min^−1^ mmHg^−1^; *n* = 6) VC responses to handgrip exercise. Data are represented as means ± standard deviation. Data were evaluated with two‐way repeated measures analysis of variance. *Significant difference (*P < *0.05) between PL and AO conditions in the low VC group.

In the PTSD group, an exploratory analysis was performed employing a median split of VC responses to examine the impact of AO supplementation in individuals possessing either low (3 kg: ≤1.0 mL min^−1^ mmHg^−1^; 6 kg: ≤1.5 mL min^−1^ mmHg^−1^; *n* = 7) or high (3 kg: >1.0 mL min^−1^ mmHg^−1^; 6 kg: >1.5 mL min^−1^ mmHg^−1^; *n* = 6) VC responses to handgrip exercise. This analysis revealed a significant group (low vs. high VC group) × condition (PL vs. AO) interaction effect (*P* < 0.01; Figure 2a) for both the 3 kg (*P* < 0.01; Figure 2a) and the 6 kg (*P* < 0.01; Figure 2b) workload. A *post hoc* analysis identified a significant difference between conditions with a significant increase in ΔVC following AO supplementation in the low VC group (3 kg: *P* < 0.01; 6 kg: *P* < 0.01), but not the high VC group (3 kg: *P* = 0.32; 6 kg: *P* = 0.23). Analysis of MAP responses in the two VC groups revealed significant group × condition interaction effects for both the 3 kg (*P* = 0.01; Figure 2c) and the 6 kg (*P* < 0.01; Figure 2d) workload, with *post hoc* analysis revealing significant decreases in ΔMAP responses following AO supplementation in the low VC group only (low VC: 3 kg: *P* = 0.01; 6 kg: *P* < 0.01; high VC: 3 kg: *P* = 0.28; 6 kg: *P* = 0.35). Further analysis of BF responses in the two VC groups revealed a significant group × condition interaction effect in the 3 kg workload (*P* = 0.05; Figure 2e), but not the 6 kg workload (*P* < 0.01; Figure 2f), but a subsequent *post hoc* analysis of the 3 kg workload did not reveal any significant changes in ΔBF responses following AO supplementation in either group (low VC: *P* = 0.20; high VC: *P* = 0.10). Group main effects were observed across all workloads and variables and the low VC group displayed significantly lower VC and BF responses and significantly greater MAP responses during handgrip exercise when compared to the high VC group (Figure 2). The lone exception was the ΔVC response at the 6 kg workload (Figure 2b), which reported no group main effect, but a significant main effect of condition with the AO condition revealed to be significantly greater than the PL condition.

No significant correlations were revealed when examining PCL‐5 scores with outcome variables or participant characteristics (e.g., moderate and vigorous physical activity, body fat %, maximum handgrip workload achieved) within the PTSD group.

## 4 DISCUSSION

The purpose of this study was to investigate whether the previously reported upper limb microvascular dysfunction and sympathetic nervous system predominance observed at rest in young individuals with PTSD impact peripherally mediated responses during handgrip exercise and, if negatively altered, these responses could be restored following acute AO supplementation. A major finding of this study was that exercise‐induced changes in VC were significantly lower in young individuals with PTSD when compared to age‐ and sex‐matched controls. Following acute AO supplementation in the PTSD group, VC was elevated to a level to which they were no longer significantly different from controls, suggesting the role of redox imbalance in mediating vascular dysfunction in PTSD. An exploratory analysis of individuals with PTSD separated by low and high VC responses to handgrip exercise revealed that the AO supplementation was most effective at improving exercise‐induced VC responses in PTSD participants displaying the lowest VC responses, a finding that was further revealed to be driven by blunting of the elevated exercise‐induced MAP responses in this group. Taken together, this study revealed abnormal, peripherally mediated exercise responses in young individuals with PTSD that may be linked to a redox imbalance or redox‐sensitive vascular/autonomic mechanism.

### 4.1 Abnormal vascular conductance responses to handgrip exercise

Prior work by our group identified lower microvascular function in the upper limb as well as SNS predominance at rest in young individuals with PTSD when compared to controls matched for age, sex and physical activity level (Weggen et al., 2021). The current study further examined whether these impairments in physiological processes are evident during exercise, a tightly regulated state that relies on adequate vascular function and precise autonomic nervous system signalling to adequately perfuse metabolically active muscle. The major finding of this study was that young individuals with PTSD displayed significantly lower VC during handgrip exercise (vs. controls). Lower vascular conductance can indicate an inability to effectively perfuse working tissue in the face of substantial peripheral vascular dysfunction, inappropriate SNS activation, and/or inadequate buffering of SNS activation in response to the exercise stimuli (Grotle et al., 2020). Therefore, the finding of significantly lower exercise‐induced VC (vs. controls) provides additional evidence of vascular and/or autonomic dysfunction in this population as well as highlighting further evidence of negatively altered cardiovascular function in young individuals with PTSD. Of interest, this current study revealed that, on average, the PTSD group reported no significant difference in exercise‐induced pressor responses when compared to the control group, a finding that was contrary to the study hypothesis. The study hypothesis was based on the current research in PTSD, which, although limited, implicates the presence of elevated exercise‐induced blood pressure responses. Indeed, two recent studies conducted in middle‐aged women with PTSD identified an amplified early onset neural and exercise pressor response, with higher muscle sympathetic nerve activity (Yoo et al., 2020) and altered action potential firing patterns (D'Souza et al., 2022) in response to isometric handgrip exercise. The mechanisms behind this elevated exercise pressor response in individuals with PTSD may be due to sensitization of mechanoreceptors (D'Souza et al., 2022) and/or adrenergic receptors, as well as compromised baroreflex function (Fonkoue et al., 2018; Park et al., 2017).

An amplified blood pressure response during exercise is predictive of hypertension (Singh et al., 1999), cerebrovascular and cardiovascular events (Allison et al., 1999; Schultz et al., 2017), and mortality (Mundal et al., 1996). Individuals at risk or with chronic medical conditions such as a history of CVD (Sandberg et al., 2018), hypertension (Sausen et al., 2009), chronic kidney disease (Downey et al., 2017), metabolic syndrome (Miyai et al., 2013) and diabetes (Karavelioglu et al., 2013) have shown an amplified exercise‐induced MAP response thought to be related to increased sympathetic activity and vascular dysfunction (Teixeira & Vianna, 2022). The current study deviates from these findings as the PTSD group did not display significantly elevated MAP responses to exercise when compared to controls. The prior studies that have identified the presence of an exacerbated exercise pressor response or highlighted potential mechanisms linked to an elevated pressor response were completed in middle‐aged and older adults with PTSD (D'Souza et al., 2022; Yoo et al., 2020). It is possible that this maladaptation is not a consistent characteristic in young individuals with PTSD, who may possess less time since the onset of PTSD symptoms and/or are less impacted by the combination of PTSD symptomology and the effects of advancing age.

### 4.2 Improved vascular conductance responses following acute antioxidant supplementation

PTSD has been linked with a significant redox imbalance due to elevated oxidative stress that is likely derived from repeated, robust increases in sympathetic nervous system activity (hyperarousal) (Hartwig et al., 2020) and hypothalamic–pituitary–adrenal axis activation (Spiers et al., 2015). A major finding of this study was that following AO supplementation, VC responses to handgrip exercise were enhanced in the PTSD group to an extent to which they were no longer significantly lower when compared to the healthy controls. Further, an exploratory analysis within the PTSD group only revealed that AO supplementation substantially improved the VC responses to handgrip exercise in those within the PTSD group who reported the lowest exercise‐induced VC responses. Further analysis revealed that these improvements following AO supplementation were driven by improving exercise‐induced MAP, but not BF, responses in the low VC group. This finding may implicate redox imbalance as a mediator for the lower exercise‐induced VC responses observed in the current study. Indeed, redox imbalance can act to directly or indirectly neutralize numerous vasodilatory molecules and increase the production of circulating and endothelial‐derived vasoconstrictors resulting in peripheral vascular dysfunction (Wray et al., 2017). Elevated pressor responses during exercise can result from substantial peripheral vascular dysfunction, as augmented MAP serves to augment limb perfusion pressure to overcome any inadequate limb perfusion during exercise (Grotle et al., 2020; Hogwood et al., 2023). In support of this, prior work by our group identified lower microvascular function in the upper limb of young individuals with PTSD (Weggen et al., 2021), providing evidence of peripheral vascular dysfunction as a potential mediator of this augmented exercise‐induced MAP response observed in some, but not all, participants with PTSD. Alternatively, redox imbalance can also exacerbate normal SNS‐mediated responses via sensitization of skeletal muscle afferents (group III/IV afferents) (Koba et al., 2013) and adrenergic receptors (Girouard & de Champlain, 2005) potentially leading to greater SNS responses for a given stimulus and greater pressor responses to given amounts of SNS activity, respectively. Independent of the direct effects of redox imbalance on vascular conductance during exercise, antioxidant supplementation may have improved the production of vasodilatory molecules through enhanced enzyme coupling. Indeed, vitamin C, a component of the antioxidants given in the current study, is well accepted as a molecule that can recycle critical components of the endothelial nitric oxide synthase enzyme allowing for more enzyme coupling and nitric oxide‐induced vasodilatation (Baker et al., 2001). Further research is needed to determine if these augmented blood pressure responses to handgrip exercise observed in some, but not all, young individuals with PTSD were mediated by SNS overactivity, elevated blood pressure responses due to perfusion limitations secondary to peripheral vascular dysfunction, or independent of both via antioxidant‐induced elevations in vasodilator production/bioavailability. Of note, severity of PTSD (assessed via the PCL‐5) did not correlate with any of the major outcome variables, specifically exercise‐induced VC or MAP responses. Further study is warranted to determine if other factors such as time since diagnosis and PTSD trauma type plays a fundamental or additive role in these maladaptations.

### 4.3 Experimental limitations

This study retroactively selected the control group, enabling us to provide an age and sex‐match comparison, but this method did not allow for the control participants to be assessed for PTSD using the PTSD Checklist (PCL‐5). All control participants self‐reported being free of medical and mental health issues, which indicated that they were not diagnosed with PTSD. To maximize the participation and adherence of those with PTSD, the study visits for females in the PTSD group were not confined to the early follicular phase of their menstrual cycles, as the female participants in the control group were. Previous studies have suggested that oestrogen has a favourable influence on vascular function (Baker et al., 2016; Limberg et al., 2010); however, work from our group (Weggen et al., 2023) and others (Restaino et al., 2022) has reported no significant alterations in VC, MAP and vascular reactivity during progressive handgrip exercise when evaluated between phases of the menstrual cycle. Although the PTSD group comprised the same participants who participated in a recently published study (Weggen et al., 2021), some differences did arise when data were contrasted with the prior publication. Specifically, resting HR values were significantly lower when measured prior to handgrip exercise (67 ± 12 beats/min) than at the onset of the study (88 ± 6 beats/min). It is unclear if this discrepancy was due to the extended time spent supine prior to the HG assessment (∼1.5–2 h), changes in body temperature over time, and/or reduction in anticipatory/baseline stress as the assessment continued. The six participants in the PTSD group who were prescribed medication for their psychological symptoms were not asked to stop their treatment. The most‐used medication (*n* = 4) were serotonin reuptake inhibitors (SSRIs), which may be beneficial to vascular function (Dimoula et al., 2022) and do not appear to alter blood pressure in young adults (Romańczyk et al., 2021; Zhong et al., 2017). Though no evaluation of the presence of depression or anxiety was administered, it is well established that these mental disorders are highly comorbid in individuals with PTSD (Ginzburg et al., 2010; Spinhoven et al., 2014). Major depressive disorder has been associated with oxidative stress‐related microvascular dysfunction (Greaney et al., 2019), while no such relationship with anxiety has been identified to our knowledge. Therefore, additional research is needed to further elucidate the mechanisms and role of comorbid conditions that may underlie the abnormal exercise responses observed in this study. No other variables outside of the data provided by the PCL‐5 such as trauma type, time since the trauma occurred (length of the disorder), or number of exposures to trauma were assessed. Future research into the effect of PTSD trauma type/length and severity of symptomology on CVD risk is needed to evaluate the effects of these variables. Oxidative stress was not directly measured in this study to confirm the prior link between PTSD and elevated oxidative stress; therefore, it is unknown whether the impact of the AO supplementation reduced this factor by direct neutralization or through other pathways that may have improved the bioavailability of other vasoactive components. Further, no AO supplementation was employed in the control group to examine the impact of AO across groups. Although acute AO supplementation can often result in vascular dysfunction at rest (Wray et al., 2012) and during handgrip exercise (Richardson et al., 2007) in young, healthy individuals, this approach should be included in future research to better interpret PTSD‐specific findings. Finally, the contention the AO supplementation was most impactful in those with the lowest VC and highest MAP responses during HG exercise was based on an exploratory analysis within the PTSD group only. Although numerous, significant interaction effects were revealed, these findings should be interpreted with caution due to the low per group participant numbers. Thus, further study is needed to definitively determine the efficacy of AO supplementation at improving peripheral haemodynamic responses during exercise in individuals with PTSD.

### 4.4 Conclusion

This study revealed an abnormal response to peripherally mediated exercise in young individuals with PTSD, evidenced by lower VC responses during handgrip exercise than those of healthy controls. Following AO supplementation, these VC responses were augmented to a level comparable to healthy control subjects and appear to be associated with a blunting of the exaggerated exercise‐induced MAP responses present in some, but not all, young individuals with PTSD. Future research should consider the possible contribution of redox imbalance to the aberrant exercise responses seen in this population.

## AUTHOR CONTRIBUTIONS

Jennifer B. Weggen and Ryan S. Garten were involved in the conception and design of the work and all authors were involved in data acquisition. Jennifer B. Weggen and Ryan S. Garten analysed data, Jennifer B. Weggen and Ryan S. Garten interpreted the data, Jennifer B. Weggen drafted the manuscript, and all authors were involved in editing and revising the work. All authors have approved the final version of the manuscript and agree to be accountable for all aspects of the work ensuring that questions related to the accuracy and integrity of any part of the work are appropriately investigated and resolved. All persons designated as authors qualify for authorship, and all those who qualify for authorship are listed.

## CONFLICT OF INTEREST

The authors declare they have no conflicts of interest.

## Data Availability

Data can be made available upon reasonable request to the corresponding author.
